# An improved multi-view attention network inspired by coupled P system for node classification

**DOI:** 10.1371/journal.pone.0267565

**Published:** 2022-04-28

**Authors:** Qian Liu, Xiyu Liu

**Affiliations:** Business School, Shandong Normal University, Jinan, China; University of Bradford, UNITED KINGDOM

## Abstract

Most of the existing graph embedding methods are used to describe the single view network and solve the single relation in the network. However, the real world is made up of networks with multiple views of complex relationships, and the existing methods can no longer meet the needs of people. To solve this problem, we propose a novel multi-view attention network inspired by coupled P system(MVAN-CP) to deal with node classification. More specifically, we design a multi-view attention network to extract abundant information from multiple views in the network and obtain a learning representation for each view. To enable the views to collaborate, we further apply attention mechanism to facilitate the view fusion process. Taking advantage of the maximum parallelism of P system, the process of learning and fusion will be realized in the coupled P system, which greatly improves the computational efficiency. Experiments on real network data sets indicate that our model is effective.

## 1 Introduction

Biocomputing is computational method and model abstracted from biology. Membrane computing (also known as the P system) is a model proposed by Păun inspired by cells and tissues in 1998 [1]. The initial membrane computing model was abstracted from a cell, dividing the interior of the cell into different regions. Each region contains a large number of substances and rules, which can produce chemical reactions. With the continuous expansion of this model, three major computational models have been formed: cell-like P system [1], tissue-like P system [2] and neuron-like P system [3]. P system has been widely concerned because of its maximum parallelism. Membrane system is mainly composed of three elements: cell membranes, multiple set objects and rules. The cell membranes divide the entire system into different areas, so that the calculations within the membrane do not interfere with each other. Multiple set objects are assigned to different membranes for calculation. The implementation of rules can change the structure of the membranes or the object within the membrane and enable communication between the membranes. Since the rules in the membrane are executed independently, P system presents the characteristics of distributed and parallelism.

Inspired by mathematics, biology, computer science and other disciplines, researchers have proposed various extended P systems based on the structure, objects, rules and calculation methods within the system. In recent years, P system has made different progress in both theory and application. In theory, many variations of P system are proposed. The tissue P system with fission rule is proposed in [4]. The number of cells can increase exponentially in the process of calculation, and its computing power is verified. Song et al. also propose a tissue-like P system with a promoter [5], whose role is to regulate the execution of rules. Subsequently, the P system with proteins on the membrane is also proposed, and the execution of the rules is closely related to the proteins [6]. Due to the constraints of space filling curves, a P system variant with directed partitioning rules and external inputs is proposed, which can efficiently solve problems that may be difficult to solve [7]. Liu et al. introduce the concept of collaboration into tissue-like P system, separating the rules in each cell and calling these rules separately in the calculation process [8]. A unidirectional tissue-like P system with promoter is proposed in [9], in which cells can only communicate in one direction, proving that this system also has potential utilization.

The theoretical research of membrane computing is developing rapidly and vigorously, which promotes the design of this model in application [10]. Membrane system models are widely used in engineering optimization, power system fault diagnosis, ecosystem modeling and other aspects [11, 12]. In terms of application, many extended membrane algorithms have been proposed to solve problems and have been optimized in improving the efficiency of the algorithm and reducing the time complexity [13–15]. Based on tissue-like P system with promoters and inhibitors, an algorithm called ECTPPI-Apriori [16] is also proposed. When processing large-scale data sets, the time complexity of this algorithm is significantly better than traditional algorithms. Peng et al. propose a new variant of tissue-like P system, fuzzy tissue-like P system, which shows its accuracy in fault diagnosis of power systems [17]. Song et al. are inspired by the rules of cell division and lifted the time limit on the rules to solve the subset and problem [18]. Due to the parallelism of the P system, the time complexity of the algorithm is reduced. Jiang et al. proposes a new clustering method to calculate the density of data based on K-nearest neighbour and Shannon entropy, and introduces tissue-like P system to realize its clustering process, and verifies its effectiveness [19]. Sang et al. improve the BBO algorithm on the basis of the hierarchical tissue-like P system that triggered the ablation rules, reducing the computational complexity of the algorithm [13]. P system can be adopted in practical applications mainly because of its maximum parallelism. Rules in cells can be executed simultaneously to the maximum extent, which greatly reduces the running time and effectively improves efficiency. Since the P system has excellent potential to handle massively parallel algorithms, its implementation also needs to choose a good hardware platform [20].

In the real world, there are a large number of complex networks, such as citation network, traffic network, social network, etc. The prediction and classification of networks have aroused wide attention of scholars. In this work, we mainly study the task of node classification in network analysis. In order to solve the problem of node classification in network, network embedding methods have been widely studied. Network embedding aims to learn a set of low-dimensional vectors to represent nodes, such as Deep Walk [21], Node2vec [22], LINE [23], etc. With the development of graph analysis tasks, GNN methods are also developed to deal with node classification problems [24]. The main idea of GNN is the message transmission between nodes, that is, the information of each node and its neighbour nodes are embedded through neural network. And then the learning results are used to process classification tasks.

The purpose of graph neural networks (GNNs) is to process graph related tasks, to aggregate neighbour nodes according to graph topological structure, and to learn embedded representation. With the advantages of end-to-end training, GNNs is widely used to deal with graph domain problems. The most representative of GNNs is the graph convolutional networks (GCN) [25]. It generalizes the convolution operation to graph data, so that each neighbour takes the same weight, generating a node representation for node classification tasks. Since there may be implicit relationships between nodes in a graph, adaptive graph convolution network is proposed to learn the underlying relationships [26]. With the development of neural networks for operations on graphs, graph convolutional networks are used to process multi-relational data in knowledge bases [27]. As GCN develops, mechanisms in RNN are introduced to reduce the limitations of GNN models. The gated recurrent unit is introduced in GNN, and a generalized neural network is proposed, which has good performance in solving the graph of the output sequence [28]. The LSTM mechanism in RNN is also introduced, which utilizes Graph-LSTM to learn representations during graph propagation, showing strong representational capabilities in natural language processing problems [29, 30]. On the basis of GCN, Veličković et al. propose the graph attention network (GAT) [31], introducing the attention mechanism into the model and assigning different weights to each node to constitute the embedded learning of global information. In the semi-supervised node classification task, the introduction of GAT outperforms GCN. In addition, the gated attention network also utilizes the convolutional self-network to assign different weights to each attention head [32]. Weights have an important impact on the learning process of the network, and the choice of weights is closely related to the network and activation function [33]. These methods perform well in solving node classification problems, however, there are some shortcomings.

The above methods are used to process single-view network and cannot encode the information in multiple views. However, networks in the real world exist in a variety of nexus and constitute multi-view networks. Each sub-view network is composed of a relation, and these sub-views share the nodes in the network. A multi-view network consists of multiple sub-views and has multiple relationship types. For example, in an academic network, there can be three views: author collaboration, paper citation, and text similarity. The author cooperative network reflects the cooperative relationship between authors. The paper citation network describes a paper citing another paper. The text similarity network represents the text similarity of two papers. Each view contains different information, so we cannot rely on just one view. Existing view models use strategies such as weighted averaging, addition, and so on, but lack collaboration between views.

To solve the above problems, we propose a multi-view attention network based on coupled P system for node classification. Since each sub-view of a multi-view network has a network relationship, we need to learn them separately. In view of this, we extend the attention mechanism to multi-view networks. This mechanism is used to assign weights to nodes in each sub-view and extract features from views in the network. Since the information of each sub-view is different, we design a view fusion mechanism. In this mechanism, the attention mechanism is further extended to fuse each view learned to form a global representation. Finally, this global representation is used for the node classification task. In order to reduce the computational complexity, we also introduce P system with parallel characteristics. The whole process of the method will be implemented in the coupled P system designed by us. For multi-view network, feature extraction can be carried out simultaneously and each module does not interfere with each other, which effectively improves computing efficiency. The main contributions are as follows:

1. We propose a new multi-view network embedding method, which can extract features from multiple views at the same time and retain the information of each view to the maximum extent.
2. We explore an attention-based view fusion mechanism. The mechanism concentrates the weight on the information view and fuses all sub-views into global representation.
3. We design a new coupled P system, which integrates multi-view networks into the coupled P system to perform node classification tasks, and reduces computational complexity by executing rules in parallel.
4. The proposed method is evaluated in multiple multi-view networks, and experimental results demonstrate the effectiveness of our model.

The rest of this paper is organized as follows. In Section 2, related work is reviewed. Section 3 describes the definition of the problem and details our model. The analysis of the experimental results is presented in Section 4. Finally, we make a summary of our work and discussed the future work in Section 5.

## 2 Related work

### 2.1 Cell-like P system

**Fig 1:**
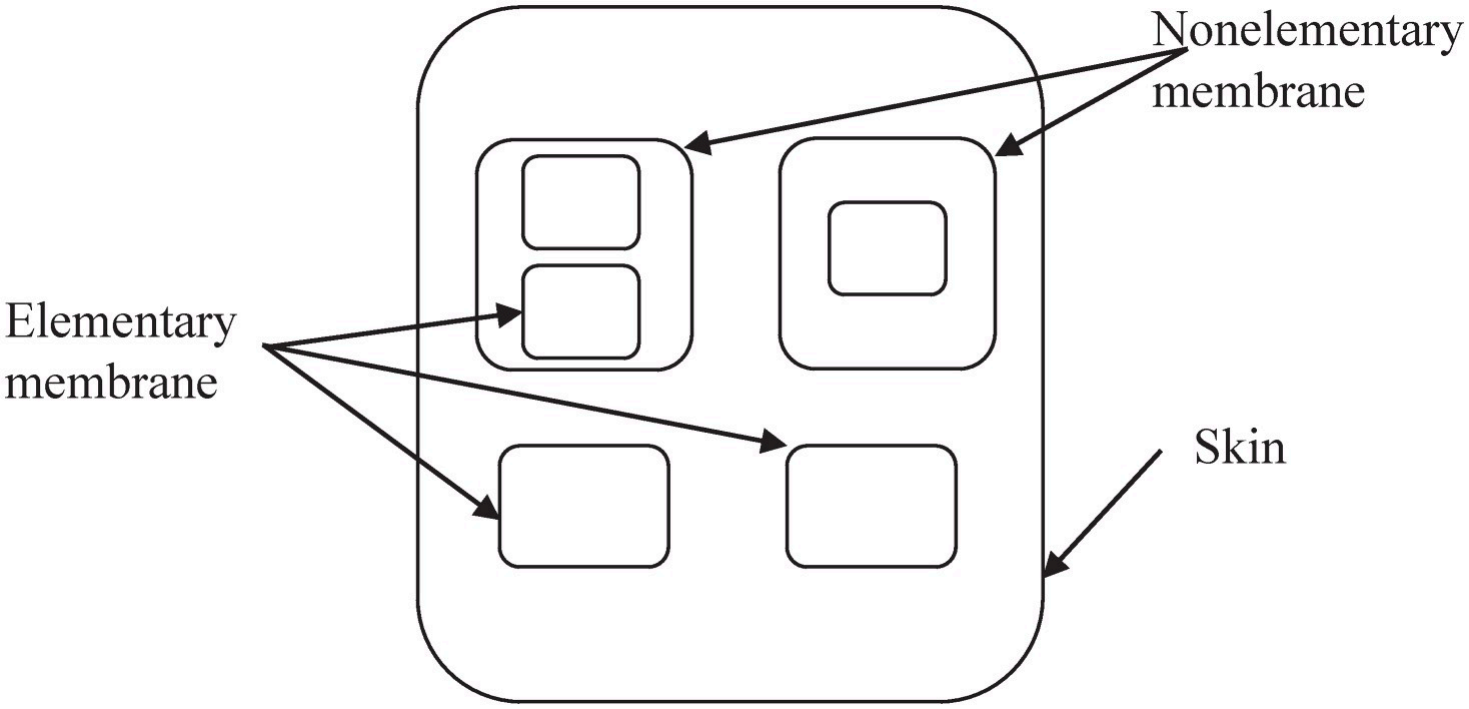
The basic structure of cell-like P system.

The cell P system is abstracted from a single cell, and the inner membrane of the cell is divided into many different regions. Its basic structure is shown in Fig 1. The outermost layer, called the skin membrane, separates cells from their environment. The innermost membrane is called the basic membrane, which means it has no other membrane structure inside it. All other membranes are called nonelementary membranes. The cell-like P system is defined as follows:

$$\begin{array}{c} \Pi =(O,u,w_{i},R_{i},i_{0}) \end{array}$$ (1)

where

1. *O* is an alphabet, and its elements are called objects.
2. *u* represents the membrane structure composed of m membranes.
3. *w*_*i*_ means the string inside membrane *i*.
4. *R*_*i*_ is the set of rules in membrane *i*.
5. *i*_0_ stands for output cell.

The P system shown above is the most basic membrane structure. There are corresponding rules in each membrane, and the rules are executed in maximum parallel.

### 2.2 Tissue-like P system

**Fig 2:**
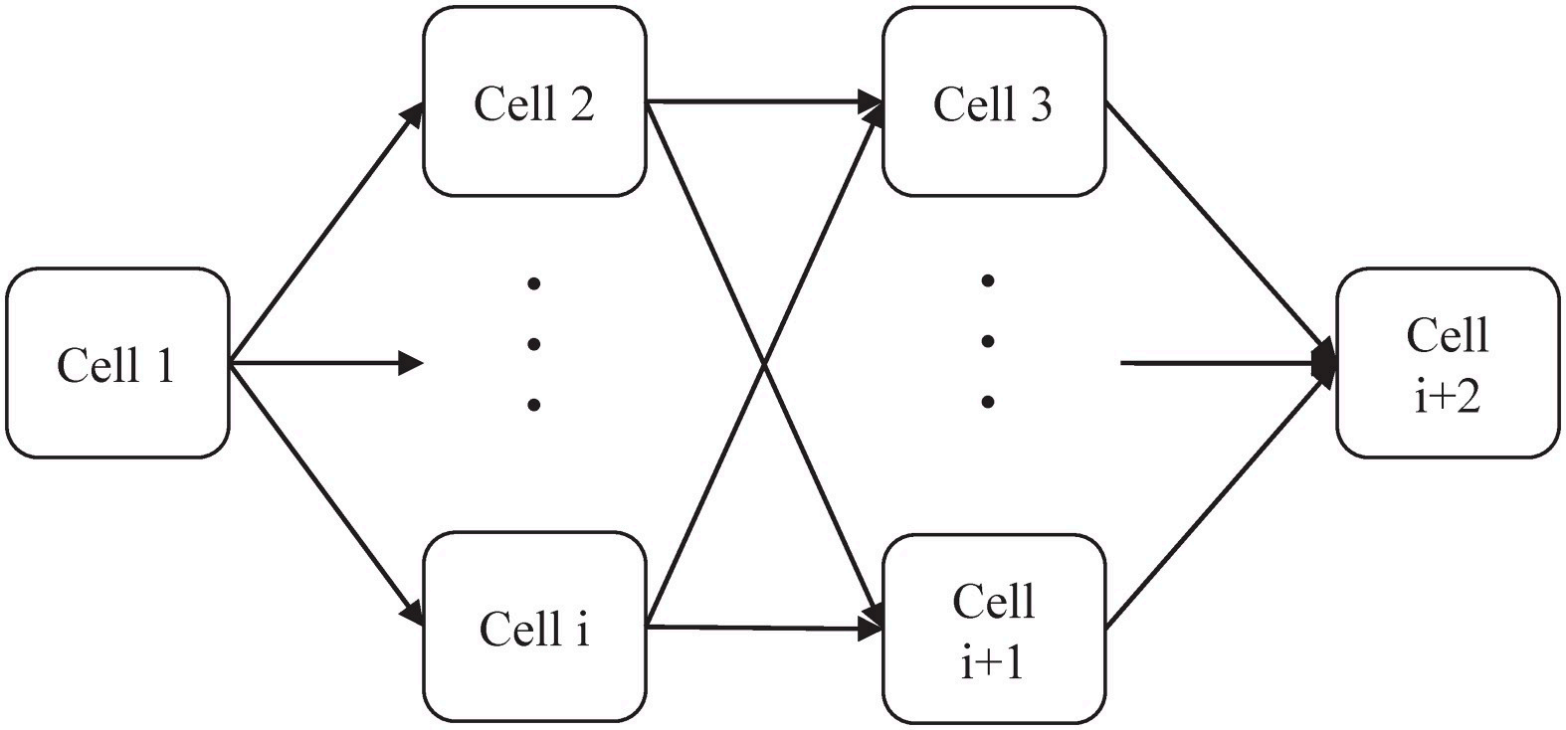
The basic structure of tissue-like P system.

Tissue-like P system is proposed on the basis of the cell-like P system. There is only one cell in cell-like P system, while tissue-like P system is composed of multiple cells [34]. Tissue-like P system also has three elements of membrane system, among which the connection between membranes plays a prominent role [35]. The basic structure of the tissue-like P system is shown in Fig 2. Cell 1 serves as the input cell and contains the initial object. Cell *i* +2 represents the output cell, storing the final result. The other cells act as intermediate cells to carry out the rules of evolution and carry out the transmission of information. The basic form of the tissue-like P system is defined as follows:

$$\begin{array}{c} \Pi =(O,\sigma_{1},\ldots ,\sigma_{m},ch,R,in,out) \end{array}$$ (2)

where

1. *O* is an alphabet set of objects in the system.
2. *σ* = {*σ*_1_, …, *σ_i_*, …, *σ_m_*} represents *m* cells in the system.
3. *ch* = {(*i, j*) ∣ *i, j* ∈ {1, …, *m*}, *i* ≠ *j*} are the set of connecting channels between cells.
4. *R* is a set of rules, including the rules of evolution within cells and the rules of communication between cells.
5. *in* stands for input cell and *out* represents output cells.

### 2.3 Network embedding

Network embedding, as a hot topic in network analysis, aims to encode complex networks into low dimensional space. Along with quantities of research on random walks, it is also referenced in network embedding. Its basic idea is to use random walk to obtain sampling path and learn embedding vector [21, 22].

In recent years, the end-to-end learning is becoming more and more popular. Due to the influence of attention mechanism in visual field, it is also introduced into neural network. Attention mechanism is a mechanism that allocates resources according to the importance of attention objects. Attention model was first used in machine translation [36] and has gradually been widely used in neural networks. With the continuous exploration of neural networks, the attention model has been further extended in graph neural networks [31]. This method adopts attention mechanism to distinguish the importance of nodes in the network, and carries on representation learning of nodes in the network. Next, we introduce the attention mechanism in graph attention network.

For each node, a coefficient *x*_*ij*_ needs to be calculated. This coefficient explains the importance of node *v*_*j*_ to node *v*_*i*_, and node *v*_*j*_ is the neighbour of node *v*_*i*_. *x*_*ij*_ = *a*(*W* ⋅ *x*_*i*_, *W* ⋅ *x*_*j*_), where *W* is a learnable weight matrix. As the attention function, *a* is a single-layer feedforward network, which is parameterized by learnable weight vector *b* ∈ *R*^2*F*^. *F* is the number of node output features. Then, we use softmax function to normalize the coefficient. $\alpha_{ij}=softmax\left(x_{ij}\right)=\text{exp}\left(x_{ij}\right)\backslash \sum_{k\in N_{i}}\text{exp}x_{ik}$, *N*_*i*_ is the neighbour set of node *x*_*i*_. The normalized coefficients are used for linear combination of node features, and the nonlinear functions are used to activate. Finally, the resulting output is the updated node, $x_{i}^{{}^{\prime }}=\sigma \left(\sum_{j\in N_{i}}\alpha_{ij}W\cdot x_{j}\right)$. Single-head attention training can make the results erratic. In order to solve this problem, multi-head is introduced. Multi-head attention is to connect the final outputs of multiple single heads of attention and average them at last. The graph attention network allocates different weights according to the differences of neighbour nodes and has good performance for network embedding, but it can only deal with single-view networks. Based on this, we extend this approach to multi-view networks.

Most of the existing graph embedding methods deal with single-view networks, but the research on multi-view networks is not perfect yet. Wu et al. propose a CGVFL algorithm for the graph classification task of multi-graph views. The method evaluates the discrimination and redundancy of all sub-views and assigns weights to them according to their importance [37]. In [38], a new algorithm is proposed for multi-view learning, which takes features in the network as network views and uses neural networks to learn. In multi-layer networks, Liu et al. also propose three methods of network aggregation, result aggregation and layer cooperative analysis to project into continuous space [39]. Network aggregation treats edges in all layers equally and then aggregates them into a network. Resulting aggregation is the embedding of different layers and then the merging of vector Spaces. The layer collaboration analysis interacts with different layers and preserves the structure of each layer. For multi-view networks, a multi-task network embedding method is also proposed to learn multi-network embeddings by forcing the information-sharing embedding of each node [40]. The method is implemented by two models. One is to embed shared information as the common embedding of all networks, and the other is to embed shared information as the consistent consensus. A multi-view attention network is proposed in [41], which is an attention-based model in which each view is learned and aggregated into a view representation through view collaboration. Huang et al. propose MT-MVGCN from the perspective of multi-task and multi-view, which extracted the information of multiple views through a multi-view convolutional network, and proposed view attention and task attention to adjust the view fusion, which was ultimately used in link prediction and node classification tasks [42]. Marco Seeland et al. propose a model based on fusing visual information. Feature extraction and encoding of multiple views are performed in convolutional neural networks, and different fusion strategies are proposed based on this information [43]. A hierarchical multi-view aggregation network based on multi-view feature space is proposed in [44]. Among the various views of the feature space constructed, views in hierarchical contexts are aggregated in terms of feature-level, location-level, and modality-level to learn a unified representation of multi-view features. However, the above method has some disadvantages. The public information in the network is adopted as the embedding of the whole network, and the information description of the whole network is lacking. Moreover, each view is treated indiscriminately, and there is a lack of analysis of differences in network views. Therefore, we not only fully extract the network information, but also treat the embedding of each view differently.

## 3 Method

### 3.1 Problem definition

**Fig 3:**
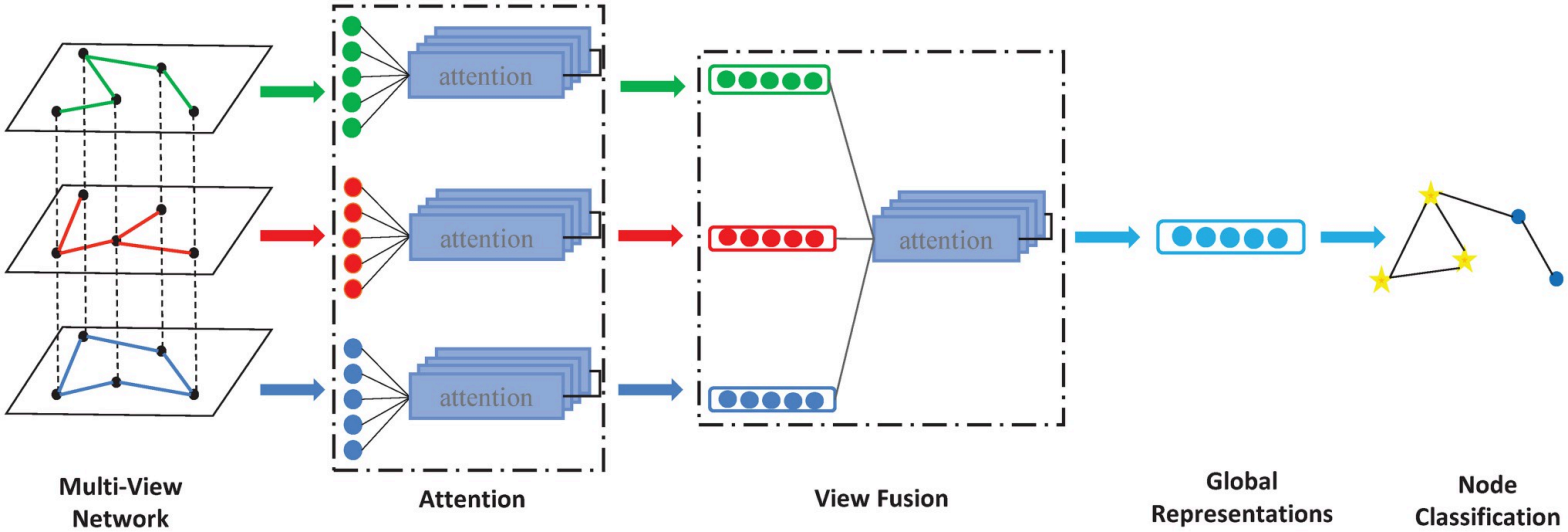
Framework of the proposed MVAN-CP model.

In this section, we introduce the proposed MVAN-CP system in detail. Firstly, we give the general framework of MVAN-CP system. Secondly, the communication and evolution rules in each subsystem are explained respectively, such as the input of multi-view network, feature extraction of multi-view attention network, view fusion and node classification. Finally, the termination conditions of the model are discussed. The notations used in this paper are described in Table 1. The paper takes the multi-view network of three views as an example, and the algorithm flow is shown in Fig 3.

**Table 1:** Description of main notations.

Notations	Description
*G*	The given multi-view network
*V*	The node set in multi-view network
*E*_*i*_	The set of edge for view *i* in multi-view network
*x*_*i*_	The feature representation of node *i* in a single view
$x_{i}^{{}^{\prime }}$	The updated representation of node *i*
*W*/*T*	The learnable weight matrix
*α*	The attention coefficient between nodes
Φ	The number of attention heads
*β*	The attention coefficient between views
*X*_*i*_	The representation of view *i*
*Z*	The final representation of the multi-view network

### 3.2 Improved multi-view attention network based on coupled P system

**Fig 4:**
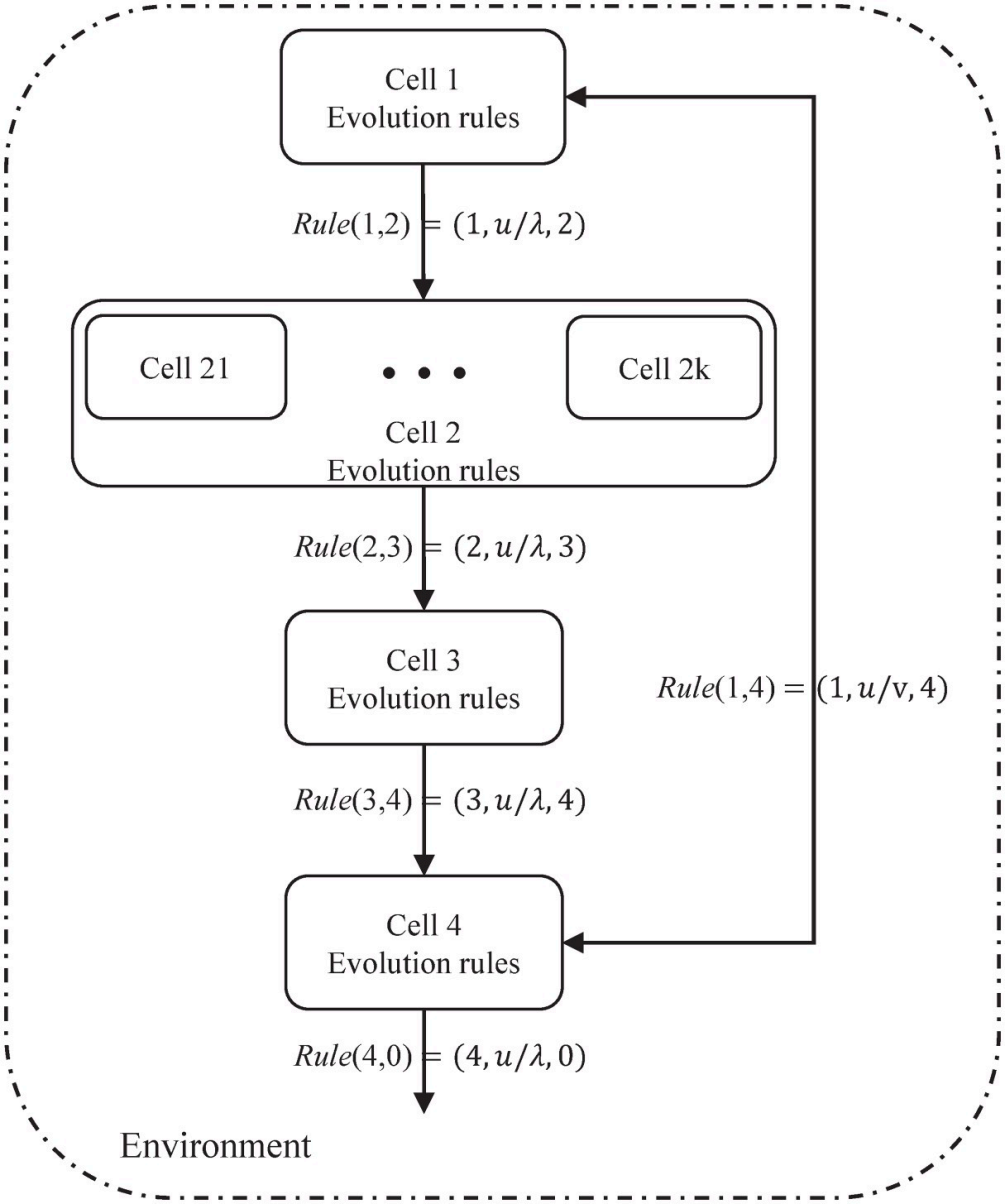
The basic structure of the coupled P system.

MVAN-CP system is proposed on the basis of coupled P system with active membrane. This section mainly discusses the design of MVAN-CP system, describes the evolution rules and calculation process of the system in detail, and expounds the communication rules between cells. We construct a coupled P system with (*K* + 4) cells, as shown in Fig 4, and its form is as follows:

$$\begin{array}{c} \Pi =(O,\sigma_{1},\ldots ,\sigma_{k+4},ch,R,in,out) \end{array}$$ (3)

where

1. *O* = {*G*, *x*_1_, *x*_2_, …, *x_V_*, *X*_1_, *X*_2_, …, *X_k_*, *Z*, *y*_1_, *y*_2_, …, *y_V_*} is a set of objects the alphabet in system. *G* is input multi-view network. *x*_*i*_ means the feature representation of node *i*. *X*_*i*_ is the characteristic representation of view *i*. *Z* represents the global feature representation of a multi-view network. *y*_*i*_ stands for the label of node *i*.
2. *σ* = {*σ*_1_, …, *σ_i_*, …, *σ*_*k*+4_ ∣ *i* = 1, …, *k* + 4} represents cells in the system, and *k* is determined by the number of views of the input multi-view network.
3. *ch* = {(*i, j*) ∣ *i*, *j* ∈ {1, …, *k* + 4}, *i* ≠ *j*} is a collection of connecting channels between cells, enabling the transfer of information between connected cells.
4. *R* = {*Ri*, *R*(*i, j*) ∣ *i, j* ∈ {1, …, *k* + 4}, *i* ≠ *j*} is a set of rules. *Ri* means the evolutionary rule in cell *i*, and *R*(*i*, *j*) is the communication rule between cells. The evolutionary rule is of the form *R* = {[*u*] → [*v*, *tar*] ∣ *u*, *v* ∈ *O*, *tar* ∈ {*here, in, out*}. The communication rule stands of the form *R*(*i*, *j*) = (*i*, *u*/λ, *j*).
5. *in* represents the input cell and *out* is the output cell.

The basic operation process of MVAN-CP system is as follows:

(1) Initialization. The multi-view network is input to Cell 1 as the input cell. All other cells are empty.
(2) Use evolutionary rule *R*1 in Cell 1. In network *G*, the sub-view and node features determined by *k* are transferred to Cell 2 by *Rule*1, and the real node labels obtained are transferred to Cell 4 by *Rule*3.
(3) *k* sub-views enter Cell 2. These views are then assigned to Cell 21, …, Cell 2k. These *k* cells have the same rules of evolution. Using the maximum parallelism of P system, the algorithm process of multi-view attention network is implemented. The feature representation of the sub-view is updated, and the updated *k* objects are transferred to Cell 3.
(4) Implement the rules of evolution in Cell 3. We use rule *R*3 to generate a global representation *Z* from *k* single views and use it to predict node labels. Finally, the node label is delivered to Cell 4.
(5) Rule *R*4 is executed in Cell 4 to calculate the loss function value between the real label and the predicted label and save the result.
(6) Repeat steps (2) to (5) until the termination condition is reached. Finally, Cell 4 sends the predicted node label with the lowest loss function to the environment.

### 3.3 The evolutionary rules

#### 3.3.1 Evolutionary rules in Cell 1

In the MVAN-CP system, the number of views in the multi-view network is determined as the first step of the whole system process. A network is made up of nodes and relationships. Ideally, there is only one relationship in a network, forming a single-view network. However, in reality, the nodes we observe usually have multiple relationships. One relationship defines one view, and multiple relationships define multiple views of the network. In order to better represent multi-view network, we introduce the basic definition of multi-view network.

**Definition 1 (Multi-view network)** Given a multi-view network with *k* views *G* = {*V*, *E*_1_, *E*_2_, …, *E_k_*}. *V* represents the nodes in the view, and each sub-view shares a set of nodes. *E_i_*(1 ≤ *i* ≤ *k*) represents the edges in the *i*-th view. Edges in each view are made up of a single relationship connected by two nodes. Sub-views with multiple sets of different relationships in a multi-view network. Cell 1 is the input cell. The original network *G* is input into Cell 1 as an object, and the network will be analysed by rule *R*1. Below we elaborate on the multi-view network analysis process in Cell 1.

$$\begin{array}{c} R1=\{\begin{array}{c} r_{11}=\{\left[G\right]\to [x_{1},\ldots x_{i},\ldots ,x_{V},out]\}\\ r_{12}=\{\left[G\right]\to [E_{1},\ldots E_{i},\ldots E_{k},out]\}\\ r_{13}=\{\left[G\right]\to [y_{i},out]\} \end{array} \end{array}$$

In Cell 1, multiple evolutionary rules are performed simultaneously to analyse the network. The number of nodes in the network is obtained by rule *r*_11_. The edge set in each view is obtained by rule *R*2, which determines that there are *k* views in the network. Rule *r*_13_ is used to obtain the real label *y*_*i*_ of each node. The above objects are transferred to Cell 2 for further evolution, and *y*_*i*_ is sent to Cell 4.

#### 3.3.2 Evolutionary rules in Cell 2

Feature extraction from the input multi-view network is a crucial step. The system proposed in this paper firstly extracts the features of a single sub-view of the multi-view network, and updates the view through the attention mechanism. The methods of obtaining attention coefficient and node update rules are described in detail below.

Before node update operation, attention coefficient should be obtained, and its calculation method is as follows:

$$\begin{array}{c} \alpha_{ij}=\frac{\text{exp}(a\left[W\cdot x_{i}||W\cdot x_{j}\right])}{\sum_{k\in N_{i}}\text{exp}\left(a\left[W\cdot x_{i}||W\cdot x_{k}\right]\right)} \end{array}$$ (4)

where ∣∣ is connection operation. *W* indicates a learnable weight matrix. A view can be expressed as *X* = {*x*_1_, *x*_2_, …, *x*_*i*_, …, *x*_*V*_}, where *x*_*i*_ is the feature representation of node *i*. Each node is updated according to Eq (5):

$$\begin{array}{c} x_{i}^{{}^{\prime }}={∥}_{\phi =1}^{\Phi }\sigma \left(\sum_{j\in N_{j}}\alpha_{ij}^{\phi }W^{\phi }x_{j}\right) \end{array}$$ (5)

where Φ is the number of attention mechanisms, and *ϕ* represents the operation performed under the *ϕ*-th attention mechanism. *N*_*j*_ indicates the set of neighbours of node *x*_*i*_, and $\alpha_{ij}^{\phi }$ represents the attention coefficient between node *i* and node *j* under the *ϕ*-th attention mechanism. *x*_*j*_ is the feature representation of the *j*-th node adjacent to the node *x*_*i*_. Within a view, we can set up multiple attention mechanisms to update nodes. For nodes obtained by multiple attention mechanisms, the average operation should be taken:

$$\begin{array}{c} x_{i}^{{}^{\prime }}=\sigma \left(\frac{1}{\phi }\sum_{\phi =1}^{\Phi }\sum_{j\in N_{j}}\alpha_{ij}^{\phi }W^{\phi }x_{j}\right) \end{array}$$ (6)

Since *k* sub-views obtained by Cell 1 are transferred to Cell 2, we update nodes after feature extraction of all views through *R*2. All sub-views in Cell 2 are assigned to *k* sub-cells. In these *k* cells, the rules of evolution are the same. Attention coefficients and node updates were obtained for all views at the same time. This process is parallel, so the computational efficiency is greatly improved. The specific evolution rules are given in *R*2.

$$\begin{array}{c} R2=\{\begin{array}{c} r_{21}=\{\left[W,x_{i},x_{j},x_{k},a\right]\to \alpha_{ij},here]\}\\ r_{22}=\{\left[\alpha_{ij},W,x_{j}\right]\to [x_{i}^{{}^{\prime }},here]\}\\ r_{23}=\{[x_{1}^{{}^{\prime }},x_{2}^{{}^{\prime }},\ldots ,x_{i}^{{}^{\prime }},\ldots ,x_{V}^{{}^{\prime }}]\to [X_{i},out]\} \end{array} \end{array}$$

In Eq (4), *W* performs a linear transformation on each node. Then it is input into a single-layer feedforward neural network a and normalized. In feedforward neural networks, weight vector *a* is usually used for parameterization, followed by *LeakyReLU* for nonlinearity. The whole process is calculated according to Eq (4), and linear transformation is performed on each node *x*_*i*_ and its neighbour nodes based on the learnable weight matrix *W*. The nodes are connected with their neighbours, and the weight vector a is used for parameterization. Finally, the attention coefficient is normalized. Different attention mechanisms can get different attention coefficients.

The nodes in Cell 2 are updated according to Eqs (5) and (6). The process is to multiply each node by the linear weight matrix *W* and the attention coefficient. Each node in the view is updated with the activation function. The update result varies according to different attention, and the final result is obtained by averaging the update result.

Output within each sub-cell is a view representation composed of the characteristics of each node, $X_{i}=\left(x_{1}^{{}^{\prime }},x_{2}^{{}^{\prime }},\ldots ,x_{i}^{{}^{\prime }},\ldots ,x_{V}^{{}^{\prime }}\right)$. Each sub-cell eventually outputs the view to Cell 2.

#### 3.3.3 Evolutionary rules in Cell 3

There are *k* views in Cell 2. However, for the implementation of subsequent tasks, collaboration between different views needs to be implemented. Therefore, the attention mechanism is further extended to capture the important information between these *k* views and merge them into one view. Below we define the view fusion.

**Definition 2 (View fusion)** We need to learn a low-dimensional representation *X*_*i*_(1 ≤ *i* ≤ *k*) for view *i*. The view fusion is to learn a function f to fuse *k* views into a global view, and the resulting view is represented as *Z* = *f*(*X*_1_, *X*_2_, …, *X_k_*).

This paper designs a view fusion mechanism that assigns different weights to views using an attention-based approach. Nodes are also assigned different weights due to different views and are eventually embedded in the global node representation. Similar to the attention coefficient of node update, the attention coefficient between views is normalized as follows:

$$\begin{array}{c} \beta_{ij}=\frac{\text{exp}(b\left[T\cdot X_{i}||T\cdot X_{j}\right])}{\sum_{j=1}^{k}\text{exp}\left(b\left[T\cdot X_{i}||T\cdot X_{j}\right]\right)} \end{array}$$ (7)

where *T* is a learnable weight matrix, and *b* represents a single-layer feedforward neural network. *β*_*ij*_ is the attention coefficient between view *i* and view *j*. The global view representation of multiple single-view networks can be obtained by using the attention gathering method, which can be defined as:

$$\begin{array}{c} Z=\sum_{j=1}^{k}\beta_{ij}\cdot X_{j} \end{array}$$ (8)

The *k* views obtained by Cell 2 are transferred to Cell 3, and the fusion of the views is performed in Cell 3. The evolution rule *R*3 is described in detail below.

$$\begin{array}{c} R3=\{\begin{array}{c} r_{31}=\{\left[T,X_{i},X_{j},b\right]\to [\beta_{ij},here]\}\\ r_{32}=\{\left[\beta_{ij},X_{j}\right]\to [Z,here]\}\\ r_{33}=\{\left[Z\right]\to [p_{i},out]\} \end{array} \end{array}$$

There are three rules in Cell 3 that need to be enforced. The rule *r*_31_ is to obtain the attention coefficient *β*_*ij*_ according to Eq (7). This rule states that the view representation is multiplied by the weight matrix and parameterized by the weight vector *b*. In order to determine the importance of view *j* to view *i*, view *j* needs to be compared with all views. The results are normalized to get the view attention coefficient *β*_*ij*_. The rule *r*_32_ is executed once the attention coefficient of the view is obtained. In this rule, *β*_*ij*_ is weighted and fused with the corresponding views to get the global view *Z*. Then we use *Z* to perform the node classification task.

The representation of the global view is also corresponding to the feature vectors of each node. After the global view is activated by the activation function, a probability of belonging to a category can be obtained. Finally, the label with the highest probability is selected as the node label. This is the result of the execution of rule *r*_33_.

#### 3.3.4 Evolutionary rules in Cell 4

Cell 3 transports the label *p*_*i*_ of predicted nodes to Cell 4 for the discrimination of node classification task. In this task, we minimize the cross-entropy loss function according to the real and predicted labels of nodes, which can be defined as:

$$\begin{array}{c} L=-\sum_{i=1}^{V}\left[y_{i}\text{log}p_{i}+\left(1-y_{i}\right)\text{log}\left(1-p_{i}\right)\right] \end{array}$$ (9)

where *y*_*i*_, *p*_*i*_ denote the real label and predicted label of node *i*, respectively. There are two evolutionary rules in Cell 4, which are described below.

$$\begin{array}{c} R4=\{\begin{array}{c} r_{41}=\{\left[y_{i},p_{i}\right]\to [L,here]\}\\ r_{42}=\{[\text{min}(L_{1},L_{2},\ldots ,L_{q})]\to [\tilde{y_{i}},out]\} \end{array} \end{array}$$

The real label *y*_*i*_ in Cell 4 is transferred from Cell 1, and the predictive label *p*_*i*_ is sent from Cell 3. *R*4 is implemented according to Eq (9). *y*_*i*_ and *p*_*i*_ are calculated after taking logarithms. When the predicted output is closer to the real label, the loss function is smaller.

$\tilde{y_{i}}$ is the final predicted node label. The loss function values obtained for many times are compared and the node label corresponding to the minimum value is taken as the final label of the node.

### 3.4 Rules of communication between different cells

Communication rules in the MVAN-CP system can be realized if there are channels between two cells or between cells and the environment. The communication rules constructed in this paper are directed communication rules to realize the communication and transmission of information between cells.

In the MVAN-CP system, there are two kinds of directed communication rules. One is one-way transmission communication rules, which exist between two cells and between cells and environment. The other is two-way transmission communication rules, which exist only between two cells.

(1) One-way transmission rule *R*(*i*, *j*) = (*i*, *u*/λ, *j*), which can transfer object *u* from cell *i* to cell *j*. Since λ is null, cell *j* cannot transfer objects to cell *i*. One-way transmission rules include *Rule*(1, 2), *Rule*(2, 3), *Rule*(3, 4) and *Rule*(4, 0).

- *Rule*(1, 2) = (1, *u*/λ 2) This rule transfers all objects obtained in Cell 1.
- *Rule*(2, 3) = (2, *u*/λ 3) It can transfer the *k* view representations contained in Cell 2 to Cell 3.
- *Rule*(3, 4) = (13, *u*/λ 4) The predicted node labels are sent to Cell 4 by this rule.
- *Rule*(4, 0) = (4, *u*/λ 0) The rule is the transfer of information between cells and the environment, which can send the final node label from Cell 4 to the environment.

(2) Two-way transmission rule *R*(*i*, *j*) = (*i*, *u*/*v*, *j*), this rule can transfer object *u* from cell *i* to cell *j*, and also transfer object *v* from cell *j* to cell *i*. Two-way transport rules include *Rule*(1, 4).

- *Rule*(1, 4) = (1, *u*/λ 4) At the beginning, Cell 1 sends the real node label *y*_*i*_ to Cell 4. After intracellular evolution, the predicted node labels and calculated loss function are stored in Cell 4. Signal the end of the calculation back to Cell 1 and repeat the whole process.

### 3.5 Termination conditions

When the system reaches the maximum number of iterations, Cell 4 takes the node label corresponding to the smallest loss function and outputs it to the environment, and the system stops running.

## 4 Experiments

In order to verify the effectiveness of MVAN-CP system proposed in this paper, we divide node classification tasks into transductive tasks and inductive tasks for experimental analysis, and present the experimental results in detail on real data sets. The experiments were conducted on a Windows 10 laptop with a 2.90 GHz CPU, 16 G RAM and a 64-bit operating system. The proposed method is compared with the previous classification algorithms, such as Deep Walk, MLP, GCN, GAT, GWNN, DGI, FastGCN, SGCN, RGCN, AEGCN and so on.

### 4.1 Evaluation indicators

In order to measure the quality of classification results, evaluation indicators are usually used to measure. In this paper, the accuracy and F1-score are used to evaluate the classification performance.

(1) Accuracy (Acc)

Accuracy is the ratio of the number of correctly classified samples to the total number of samples. It’s for the global sample, as long as the sample is correctly predicted, the numerator of the formula is added by one, and the denominator is all the predicted samples. The total number of samples is *N*, *Y*_*i*_ is the real label of the sample, *P*_*i*_ is the prediction label of the sample, and *L*_*i*_ is the number of label categories.

$$\begin{array}{c} Acc=\sum_{i=1}^{L_{i}}\frac{|Y_{i}\cap Z_{i}|}{N} \end{array}$$ (10)

(2) F1-score (F1)

The F1-score is also a measure of the classification model as a weighted average of *Precision* and *Recall*. When F1-score is larger, the performance of the model is better. The *Precision* refers to the proportion of samples of actually true to the samples of predicted to be true. The *Recall* is the percentage of samples of predicted to be true as opposed to samples of actually true. *TP* refers to the sample predicted to be positive and actually positive. *FP* is a sample predicted to be positive but actually negative. *FN* refers to the sample predicted to be negative but actually positive.

$$\begin{array}{c} Precision=\frac{TP}{TP+FP} \end{array}$$ (11)

$$\begin{array}{c} Recall=\frac{TP}{TP+FN} \end{array}$$ (12)

$$\begin{array}{c} F1=2\frac{Precision\cdot Recall}{Precision+Recall} \end{array}$$ (13)

### 4.2 Data sets

This paper utilizes four real datasets and divides them into two types: transductive learning task and inductive learning task. Transductive learning is a semi-supervised learning process during training, and the network structure and feature vectors of unlabeled nodes are known. Inductive learning means that the feature vector and network structure of the node to be tested do not exist, and the testing process is carried out on a new graph.

Three commonly employed citation networks Cora, Citeseer and Pubmed [45] are selected for experiments in the transductive learning task. In these data sets, each node represents a paper, and the citation of the paper is used as the edge. We construct two views for each data set: citation network and text similarity network. Citation networks are based on citation relationships in paper citation records. Text similarity network is constructed by cosine similarity between papers. In the inductive learning task, we used protein–protein interaction network (PPI). Only human genes are retained as nodes in the network, which has six views.

Cora [45]: This dataset is a citation network of 2708 machine learning papers that make up nodes in the network. There are 5429 edges, 7 kinds of papers and 2 views in the dataset.

Citeseer [45] The data set consists of 3312 papers with a total of 4732 edges. This network is divided into 6 categories and has 2 views.

Pubmed [45]: This dataset is a citation network of 19717 papers. There are 44338 edges, 3 types of papers and 2 views in the network.

PPI [46]: This data set is built from the STRING database V9.1. Those with human genes are preserved as nodes, with a total of 56944 nodes, more than 800000 edges, 121 protein types, and 6 views constructed.

The details of the dataset are summarized in Table 2. We use nonlinear activation function (ELU) for each data set, and the training times are 200 times. Due to different data sets, we adjust different parameters. For transductive learning tasks, we use accuracy to measure the performance of the model. For the inductive learning task, F1-score is used to measure the performance of the model.

**Table 2:** Statistics of four data sets.

Dataset	Nodes	Edges	labels	Views	Task
Cora	2708	5429	7	2	Transductive
Citeseer	3312	4732	6	2	Transductive
Pubmed	19717	44338	3	2	Transductive
PPI	56944	818716	1213	6	Inductive

### 4.3 Experimental setup

In this experiment, we compare the MVAN-CP system with the following baseline approach.

Deep Walk [21]: This is a classic network embedding model. The method uses uniform random walks to obtain information and learn a representation of the network. This method has good performance even with a small number of annotated nodes.

LINE [23]: This is a network embedding method suitable for undirected, directed and weighted arbitrary networks. It designs an objective function to preserve the first and second order approximations and proposes a breadth-first strategy to preserve the structure of the network.

Node2vec [22]: It is a method for learning the continuous representation of nodes in the network. The idea is similar to Deep Walk, but the generation mode of random Walk is improved, so that the generated random Walk retains the characteristics of depth first and breadth first.

GCN [25]: This is a method to extend convolution operation to graph data, so that all neighbours have the same weight.

GWNN [47]: In this method, wavelet transform is adopted to replace Fourier transform, so as to improve the efficiency of convolution.

DGI [48]:Based on mutual information, the model is a method to learn node representation of graph structure data in an unsupervised way.

FastGCN [49]: On the basis of GCN, the convolution process of graph is interpreted as the integral transformation of embedded function under a certain probability. The method generates node embedding by sampling vertices in a graph.

SGCN [50]: This model simplifies the structure of GCN.

RGCN [27]: This method is mainly used to process the data with highly multi-relational features in the knowledge base.

AEGCN [51]: The core of this method is still a graph convolution network. In order to reduce the loss of node-level information, autoencoders are used to constrain its hidden layer.

LGCN [52]: In terms of graph structure data, this method automatically selects a fixed number of adjacent nodes and converts the graph data into a one-dimensional network structure, so that the convolution operation can be better applied.

GraphSAGE [53]: This is a way to generate embedding for target nodes by sampling the information of nodes in the neighbourhood.

GAT [31]: In this method, attention mechanism is introduced into graph convolutional network, and different weights are learned for each node through the attention model to conduct embedded learning for network information.

GAT-Const: GAT-Const is a method of embedding representation based on GAT with constant attention weight.

### 4.4 Analysis of experimental results

**Fig 5:**
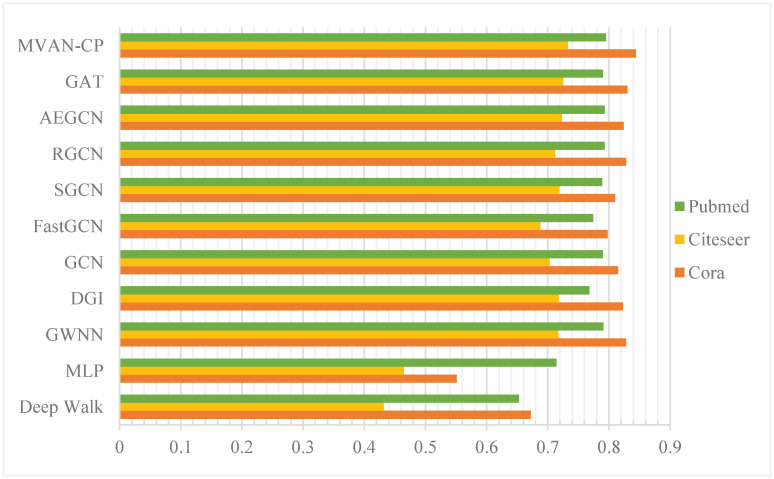
Comparison of accuracy between different methods.

The accuracy results of transductive learning in node classification tasks are shown in Table 3. Our proposed MVAN-CP performs better than other methods on all networks, including GCN and GAT. The results are even more striking on Cora networks. Based on the random walk DeepWalk, the classification results are significantly lower than the graph neural network methods. The methods of graph neural network have greatly improved the performance especially on the Citeseer dataset. SGCN, RGCN and AEGCN are all improved on the basis of GCN, and the overall experimental results are also enhanced. However, due to the introduction of the attention mechanism, we observe that the classification performance of GAT is superior to that of GCN on Cora and Citeseer networks. But the advantage in Pubmed network is not significant. With the continuous improvement of the GCN method, the performance of the improved GCN method gradually exceeds that of GAT on Pubmed network, but it is still lower than that of GAT on Cora and Citeseer network. This phenomenon shows that the attentional mechanism introduced in the task of node classification is more beneficial to node enhancement than node smoothing. MVAN-CP is based on attention mechanism and introduced into multi-view network, which consistently outperforms other methods. The accuracy comparison of each method is shown in Fig 5.

**Table 3:** Results of transductive learning in terms of accuracies.

Methods	Cora	Citeseer	Pubmed
Deep Walk	0.672	0.432	0.653
MLP	0.551	0.465	0.714
GCN	0.815	0.703	0.790
GWNN	0.828	0.717	0.791
DGI	0.823	0.718	0.768
FastGCN	0.798	0.688	0.774
SGCN	0.810	0.719	0.789
RGCN	0.828	0.712	0.793
AEGCN	0.824	0.723	0.793
GAT	0.830	0.725	0.790
MVAN-CP	**0.844**	**0.733**	**0.795**

**Fig 6:**
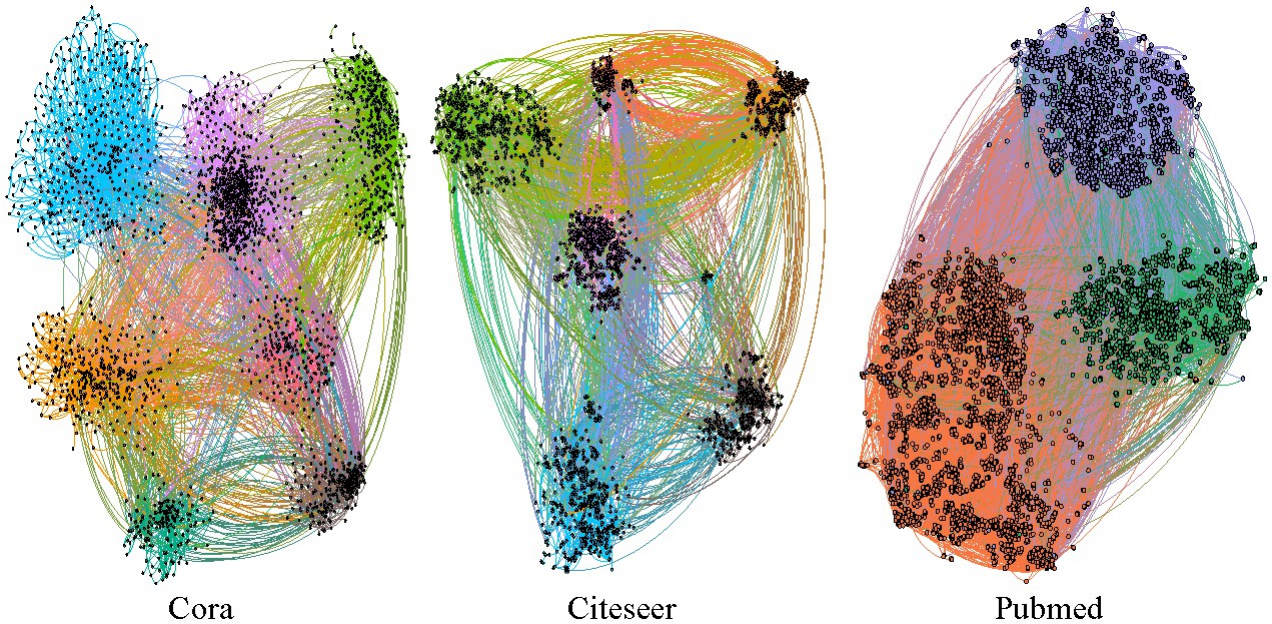
Visualization of three data sets. The colour indicates the label of the node.

As shown in Fig 6, the classification results of the above three data sets are visualized. The colour indicates the label of the node, and the classification results are different clusters. In three data sets, the system proposed in this paper can accurately divide the nodes of each label into a cluster. The MVAN-CP system performs well in node classification tasks.

**Fig 7:**
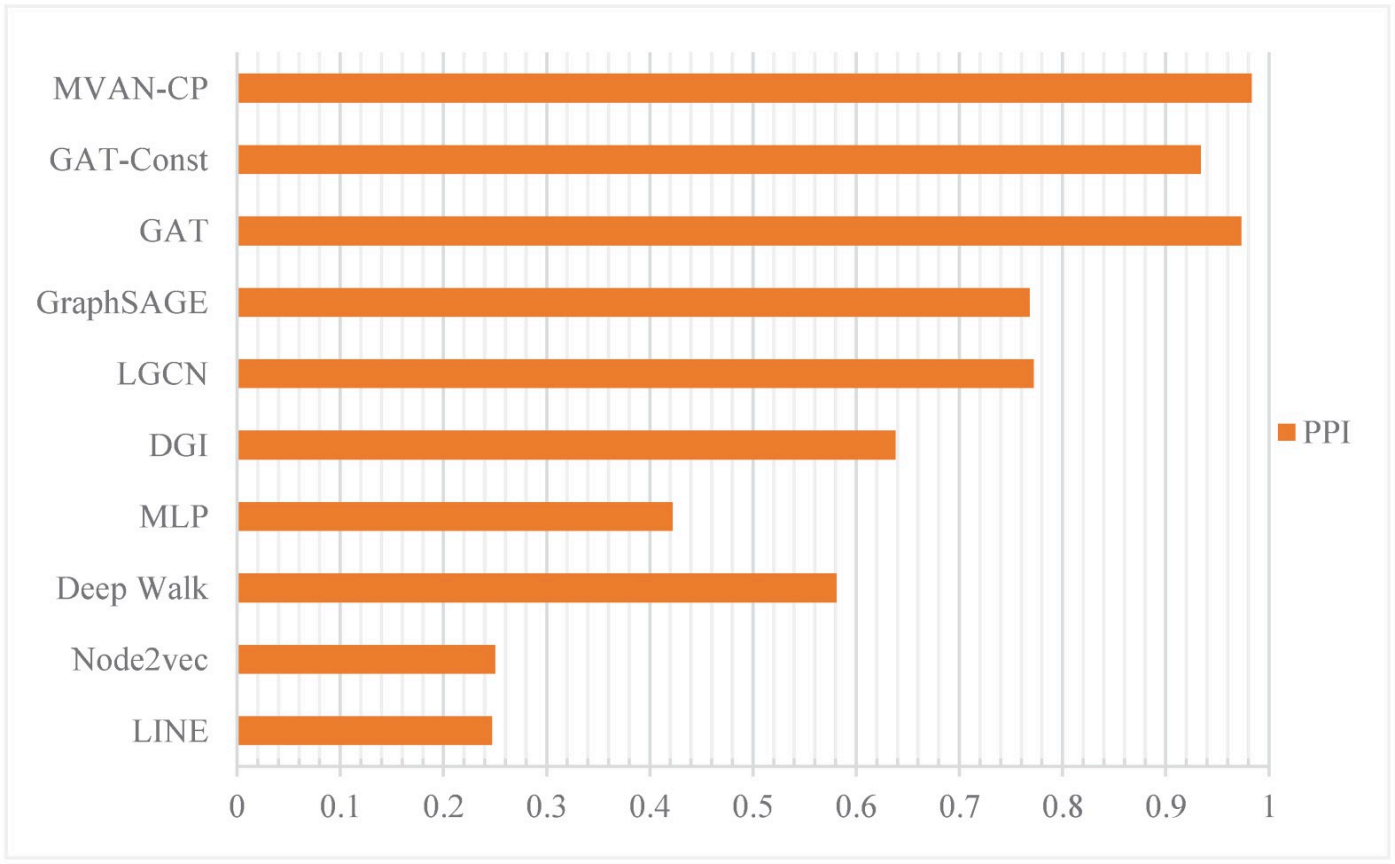
Comparison of F1-score between different methods.

The results of inductive learning in node classification tasks are shown in Table 4. It can be observed from the following table that the neural network methods are obviously superior to the traditional method. Neural network methods using the attentional mechanism have the accuracy of more than 90%. GAT-Const adopts constant attention weight, and its accuracy is lower than GAT. Therefore, the accuracy of classification results can be improved by using attention allocation weight. The results on the PPI data set are shown in Fig 7.

**Table 4:** Results of inductive learning in terms of F1-score.

Methods	PPI
LINE	0.247
Node2vec	0.250
Deep Walk	0.581
MLP	0.422
DGI	0.638
LGCN	0.772
GraphSAGE	0.768
GAT	0.973
GAT-Const	0.934
MVAN-CP	**0.983**

We compare the transductive task and the inductive task comprehensively. It can also be observed that the MVAN-CP method has more advantages in the inductive learning task than in the transductive task, which may be due to the more sufficient label information in the inductive learning task. The abundant information enables our model to better extract network features, which makes MVAN-CP have certain generality and generalization ability.

**Fig 8:**
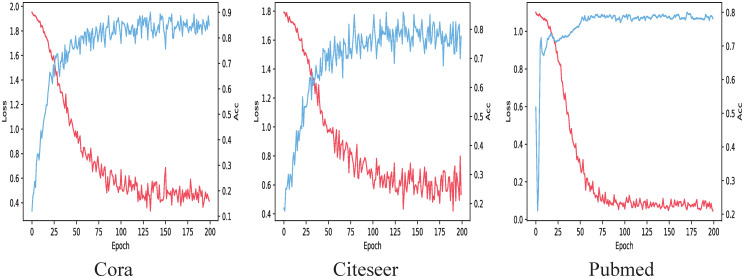
Influence of iteration times on loss function and accuracy.

The following takes the transductive learning task as an example to discuss the impact of the number of iterations on the loss function and accuracy, as shown in Fig 8. The figure below shows the changes on Cora, Citeseer, and Pubmed in sequence. When the number of iterations is less than 100, the changes of loss function and accuracy are particularly obvious. When the number of iterations exceeds 150, the changes of loss function and accuracy are relatively gentle. It just fluctuates in a small range. Therefore, we use 200 as the number of training times for the model.

**Fig 9:**
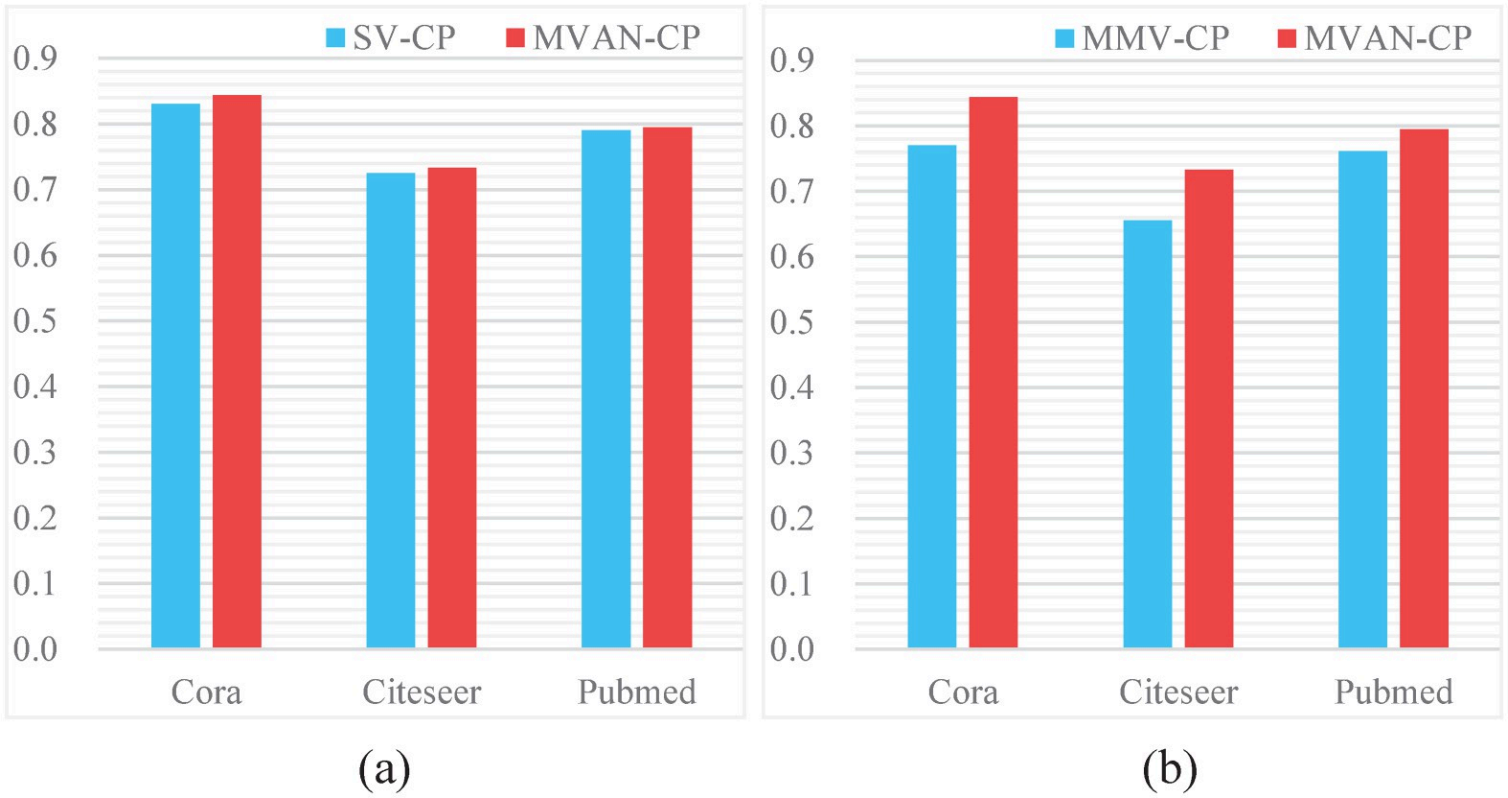
Comparison of performance of different models.

In this paper, we extend the attention mechanism, adopt multi-view attention mechanism to extract network features, and design a view fusion mechanism based on attention. The contribution of these two mechanisms to the results is assessed in this section. First of all, we do not use multiple views for feature extraction, but simply use a single view to extract features, which is named SV-CP. Secondly, the attention mechanism is not adopted in the view fusion mechanism, but the feature of each view is averaged, which is called MMV-CP. We compared these two models with MVAN-CP, and the results are shown in Fig 9.

Obviously, the designs of multiple views and view fusion have an important impact on classification task. For all datasets, performance degrades significantly after removing these two mechanisms. This phenomenon illustrates that adopting multiple views of the network can more effectively define the importance of different network relationships, and thus determine the impact on the network. In the process of view fusion, the attention mechanism focuses the weight on the view with rich information, which makes the model show good performance.

### 4.5 Parameter analysis

**Fig 10:**
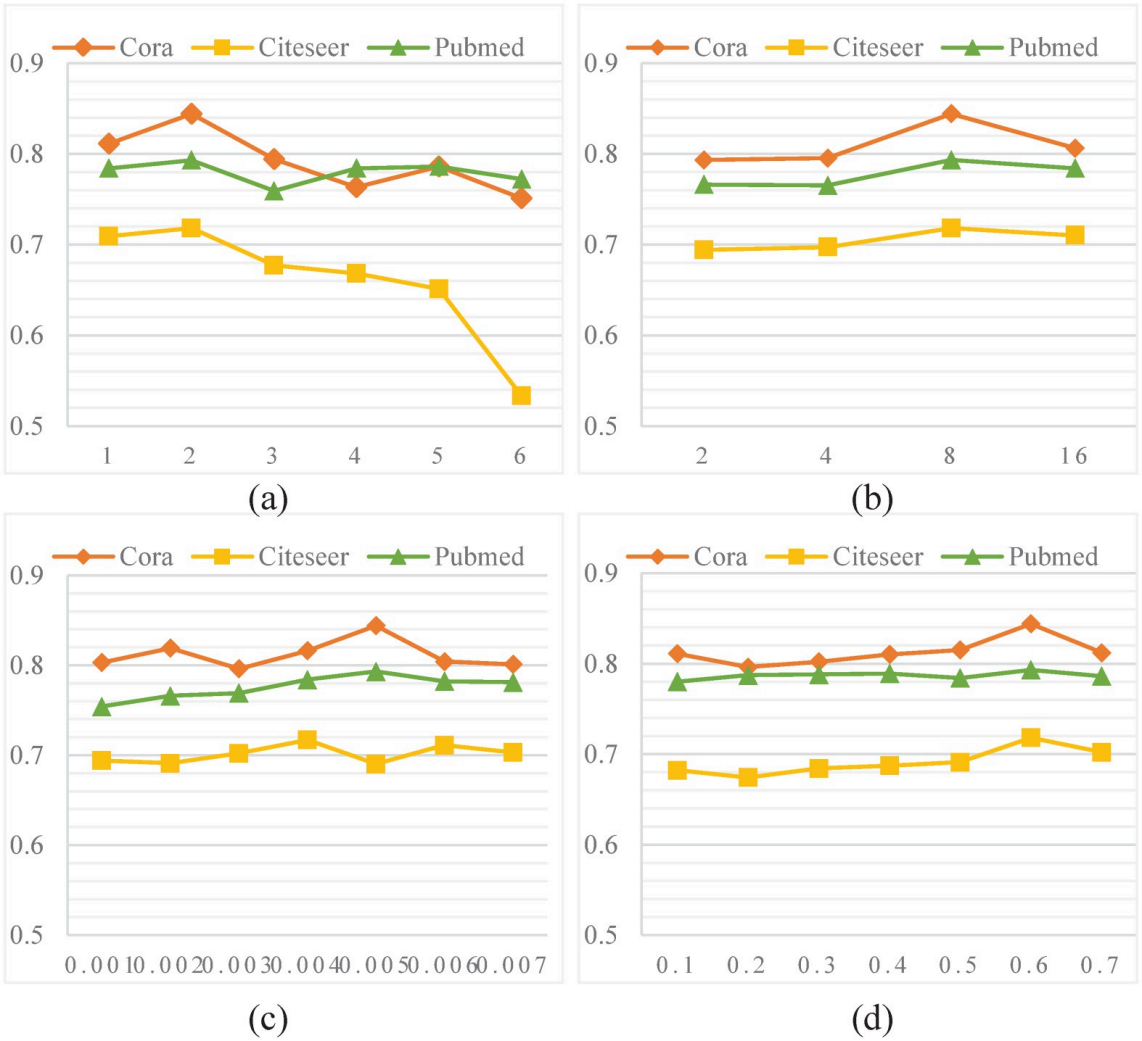
The results of parameter sensitivity experiments.

In this section, we analyse the parameter sensitivity of the proposed system. We evaluate the influence of hidden layer, attention head, learning rate and input feature dropout in node classification tasks on Cora, Citeseer and Pubmed datasets, as shown in Fig 10. All parameters have been determined except those to be checked.

Fig 10a shows the influence of hidden layer on accuracy. We set the number of layers to range from 1 to 6. For Cora and Pubmed data sets, when the number of layers is 2, the performance of node classification task is significantly better than that of other layers. With the increasing number of layers, the classification results gradually become weaker. However, for Citeseer data set, the change of hidden layer has little impact on the classification results.

The influence of attention head on node classification task is given in Fig 10b. As can be seen from the figure, at the beginning, the accuracy of the classification task increased with the increase of the number of attention head. When the attention head is 8, the accuracy is the highest. After that, the accuracy gradually decreased with the increase of attention head.

Fig 10c indicates the test result on the different learning rate. For Cora data set, accuracy fluctuate up and down with the change of the learning rate. When the learning rate is 0.005, the accuracy reaches the highest value. In Citeseer data set, the change of learning rate has little effect on accuracy. As the learning rate changes, the accuracy hardly changes much. In Pubmed data set, with the increase of learning rate from 0.001 to 0.004, we can observe the increasing accuracy. But as we continue to improve the learning rate, the accuracy gradually decreases.

The impact of input feature dropout is shown in Fig 10d. The input feature dropout range is from 0.1 to 0.7, and the fluctuation degree of the three data sets is relatively stable. When the input feature dropout is 0.6, the accuracies of all three data sets reach the highest.

**Fig 11:**
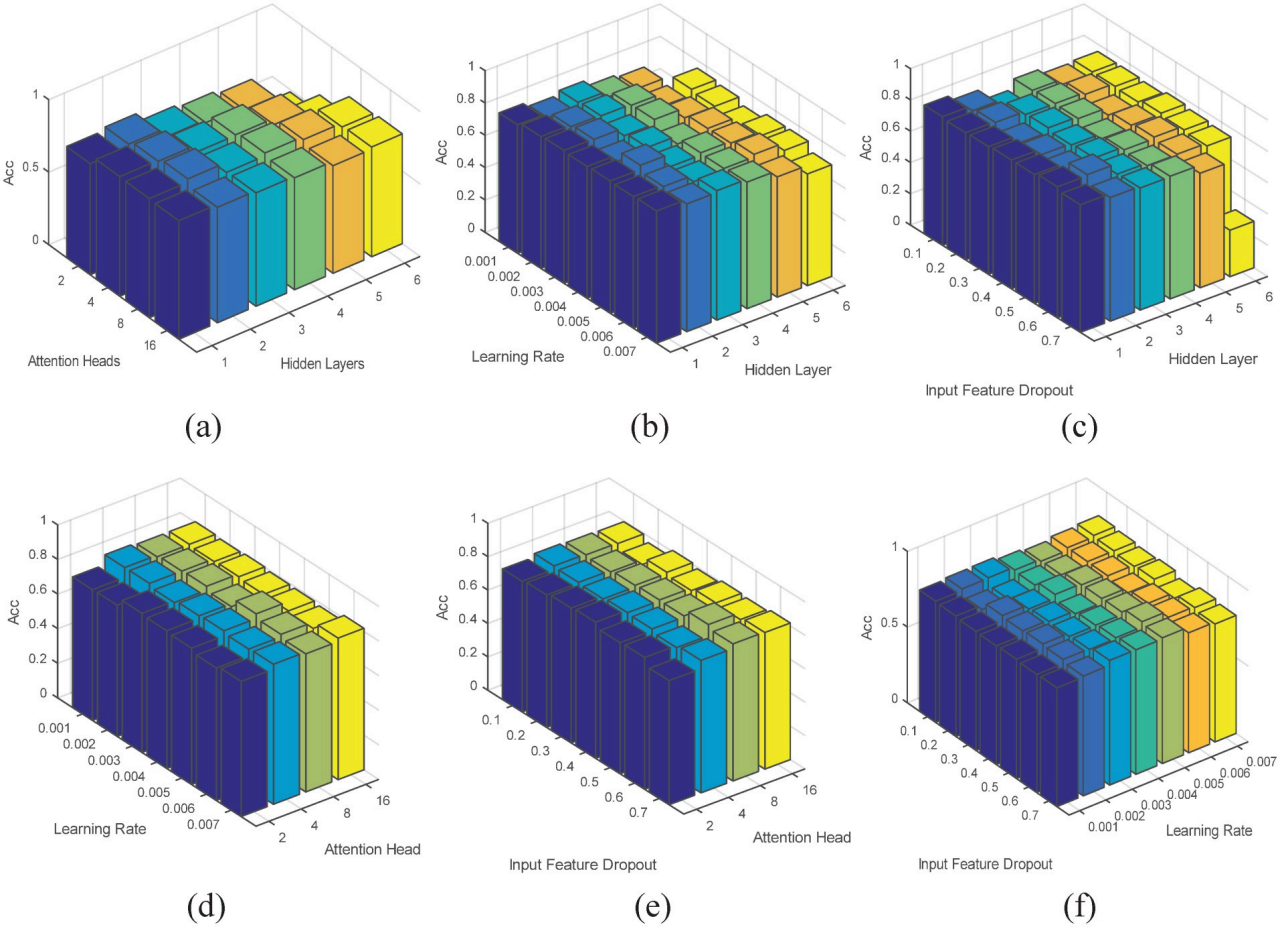
The effect of changing any two parameters at the same time on the Cora data set.

We take the Cora data set as an example to explore the impact of simultaneous changes of any two parameters on the performance of the model, as shown in Fig 11a shows the influence of the number of hidden layers and attention heads on the accuracy. It can be seen that hidden layer has a greater influence on the model. The changes of hidden layers and the learning rate are shown in Fig 11b. Fig 11c represents the variation of accuracy with the number of hidden layers and input feature dropout. Fig 11d and 11e respectively reflect the impact of changes in the number of attention heads and learning rate, the number of attention heads and input feature dropout on accuracy. It can be seen that the impact of changes of attention heads on the accuracy is more important than that. Fig 11f illustrates affecting accuracy by changing learning rate and input feature dropout. The comparison between the two shows that the role of learning rate is more important. On the whole, changing any two parameters at the same time has little effect on the model, indicating that the model has good stability. But when the number of hidden layers is 2, the number of attention heads is 8, the learning rate is 0.005, and the input feature dropout is 0.6, the classification effect of the model is the best.

## 5 Conclusions

This paper proposes a novel multi-view attention network based on coupled P system, named MVAN-CP. More specifically, we explore an attention-based mechanism for learning multi-view networks. The whole process is implemented in the coupled P system, and the efficiency of the algorithm is greatly improved by the parallelism of the P system. Firstly, the information of each node in the network is extracted based on attention mechanism, and the neighbour information is aggregated to obtain the feature representation of multiple sub-views in the network. Then, a view fusion mechanism is designed to fuse multiple views into one view by assigning attention weights according to the importance of each view to the task. Finally, the obtained learning representation is used for the node classification task. The proposed method is validated in both transductive learning and inductive learning tasks, and a large number of experiments are carried out with four data sets. Experimental results show that the proposed model is superior to existing methods in node classification tasks.

In the future, we intend to extend this model to more complex graph networks, and to deal with link prediction, recommendation and other tasks. In addition, the performance of the algorithm will be further optimized to improve the efficiency of the algorithm.

## Supporting information

S1 Raw images The original figure in the paper. (PDF)

S1 File Datasets used in experiments. (ZIP)
