# Supplementary figures and images for: An improved multi-view attention network inspired by coupled P system for node classification
Source: PLoS One. 2022 Apr 28;17(4):e0267565. doi: 10.1371/journal.pone.0267565 (PMC9049499; doi:10.1371/journal.pone.0267565)

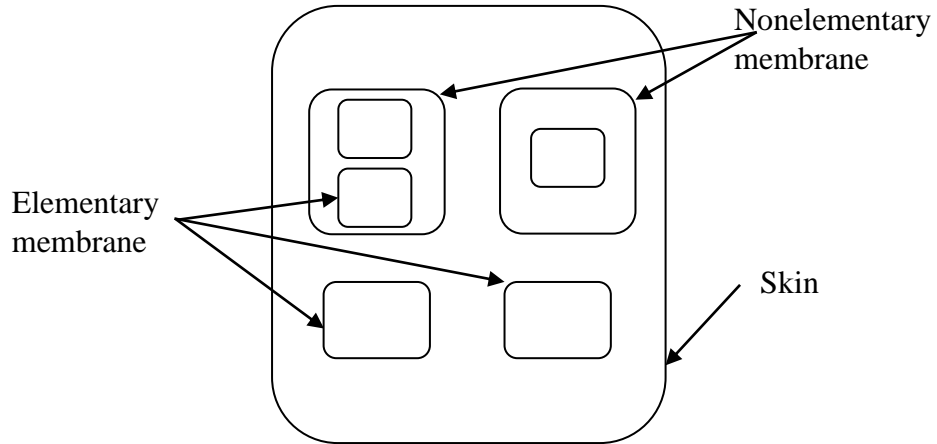

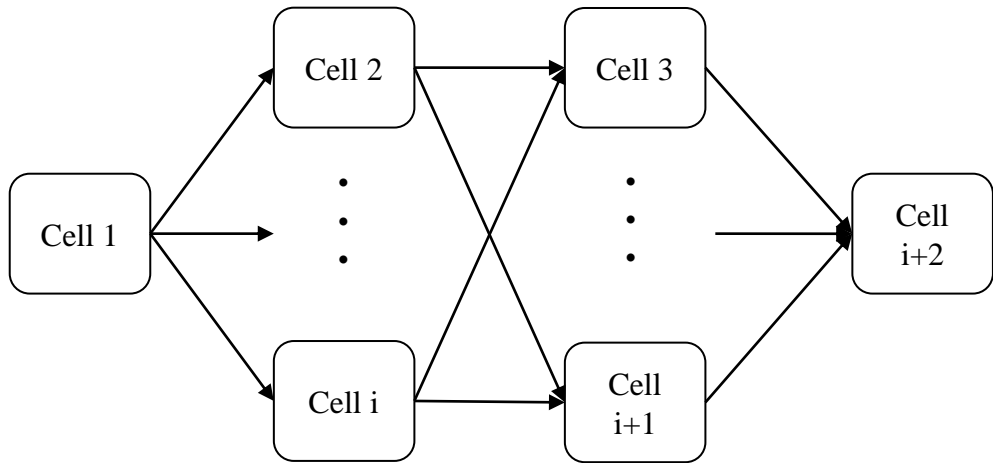

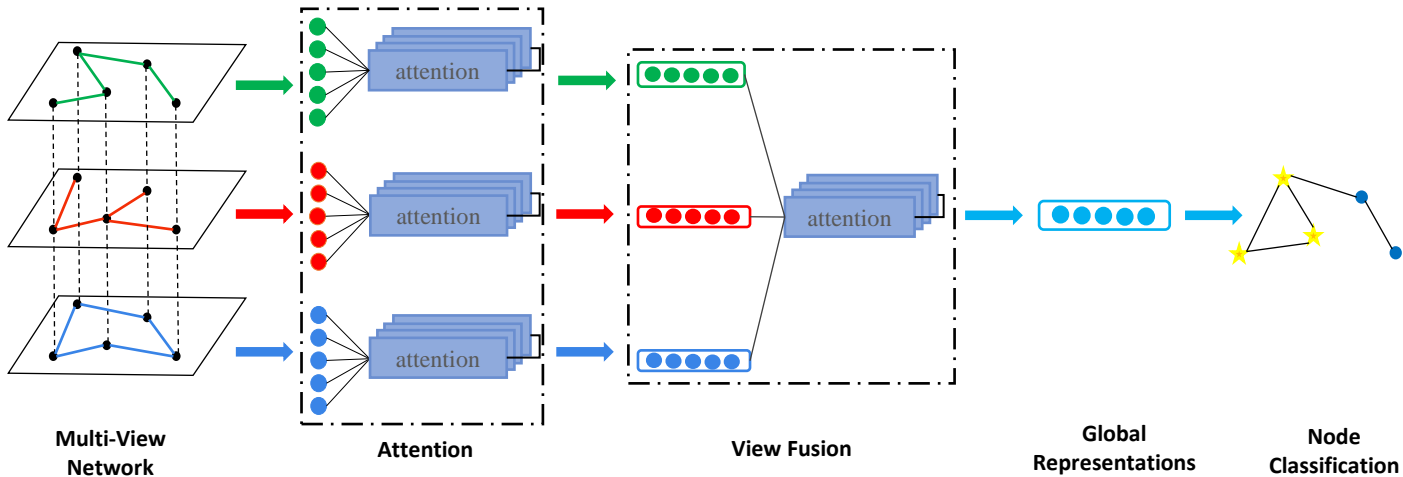

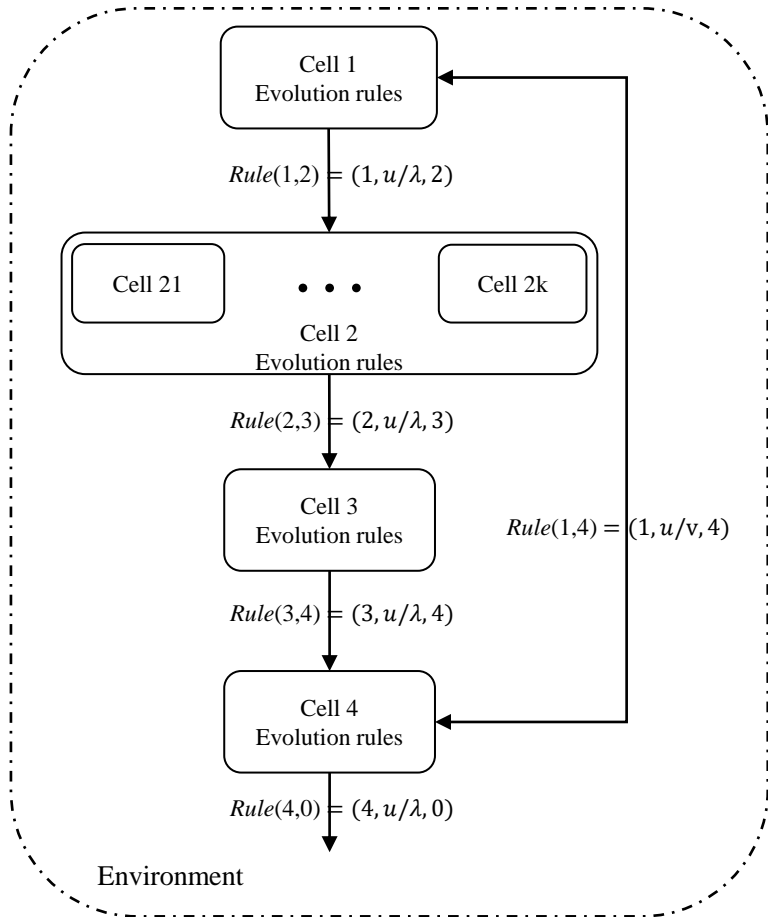

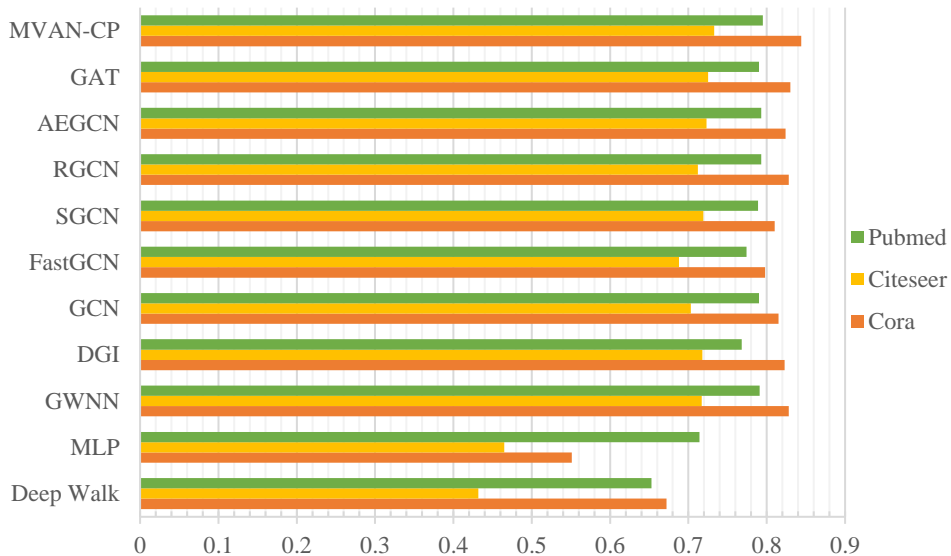

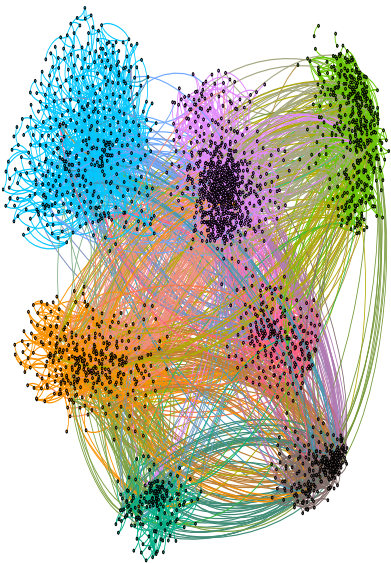

Cora

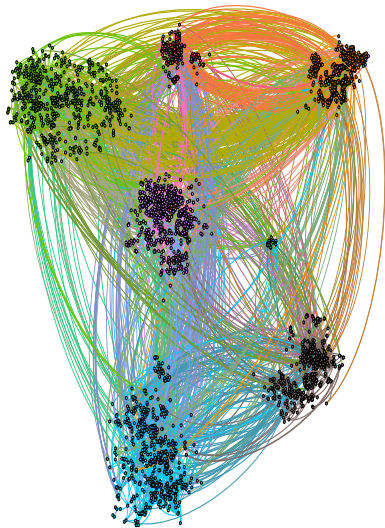

Citeseer

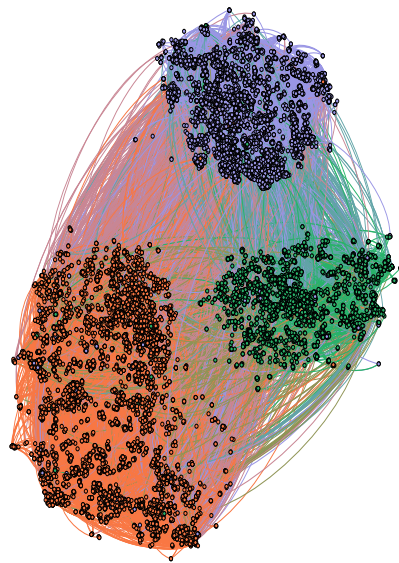

Pubmed

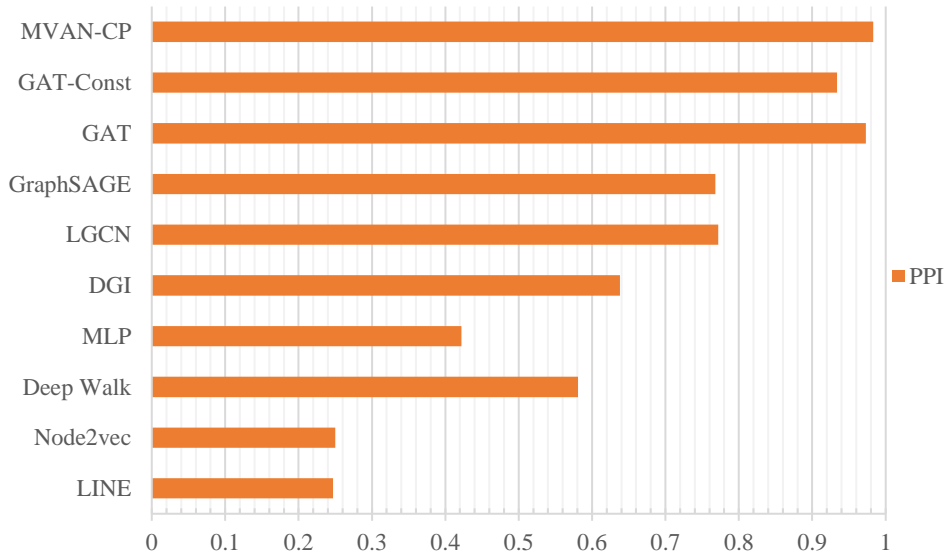

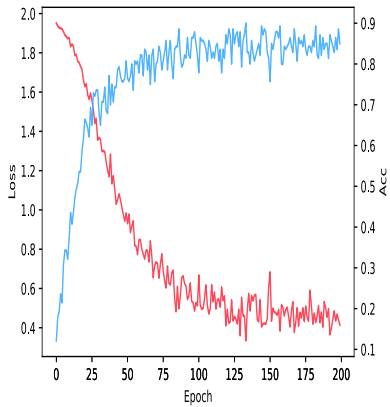

Cora

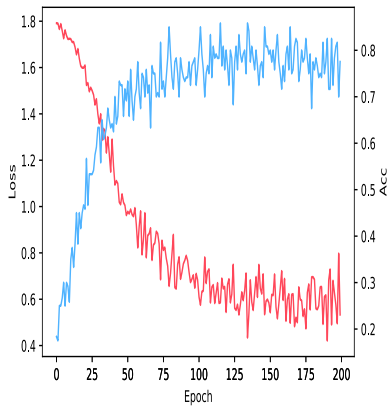

Citeseer

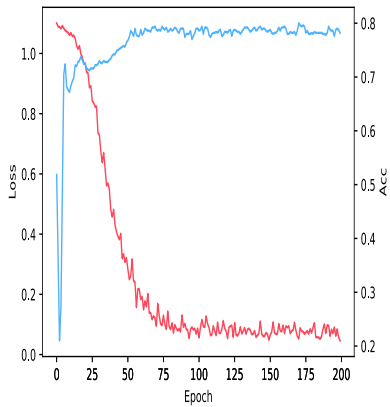

Pubmed

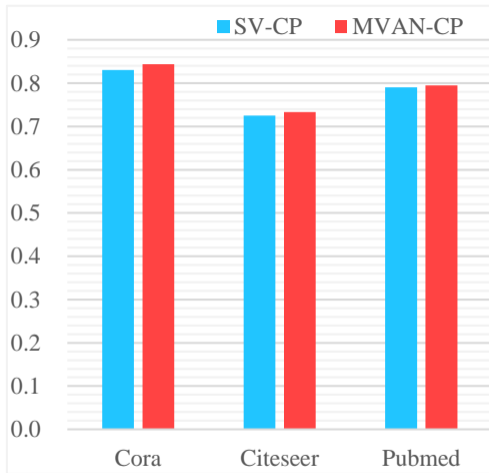

(a)

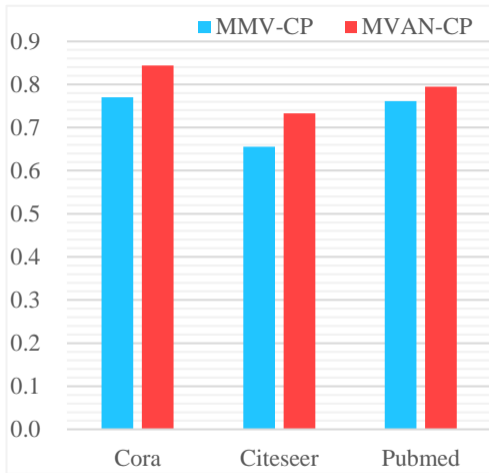

(b)

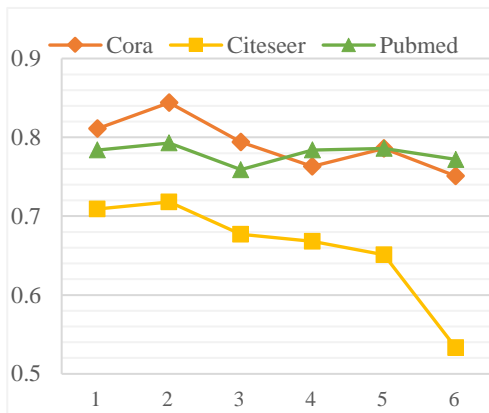

(a)

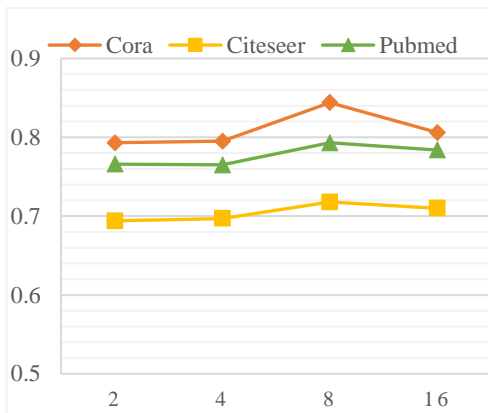

(b)

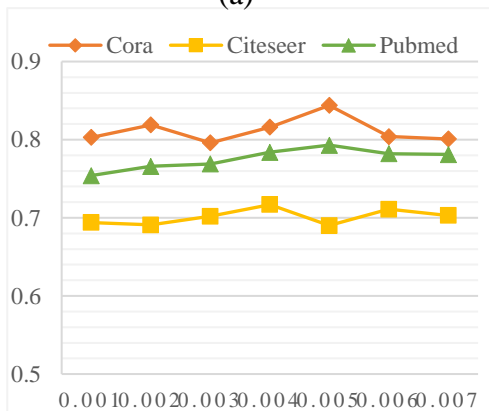

(c)

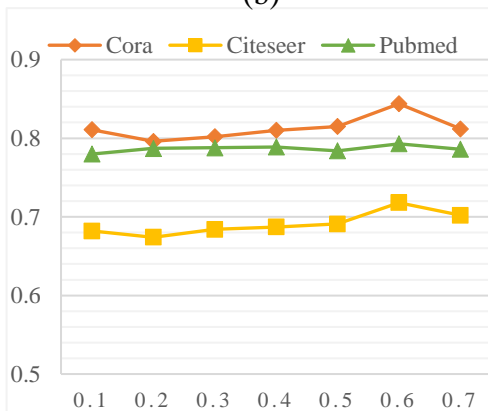

(d)

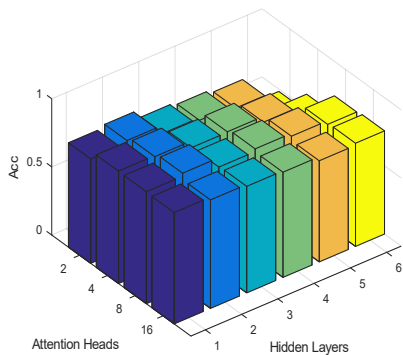

(a)

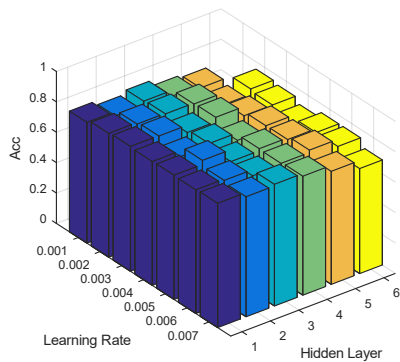

(b)

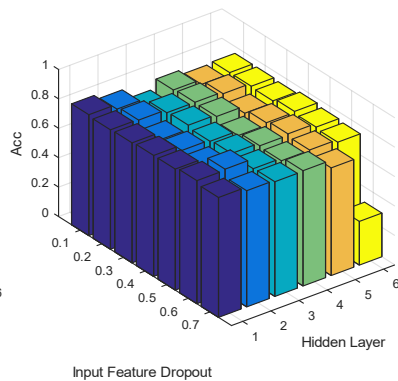

(c)

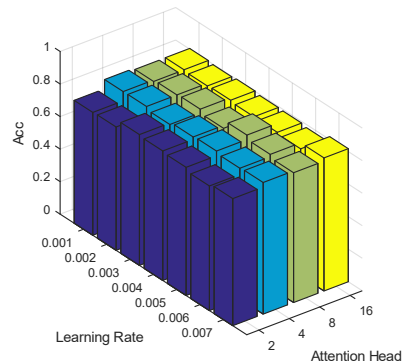

(d)

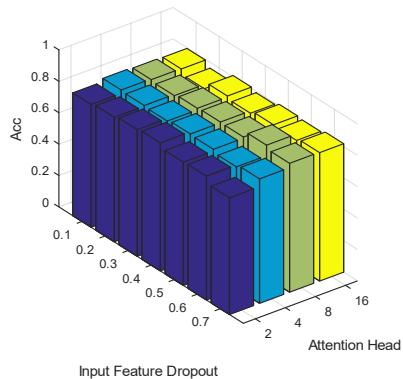

(e)

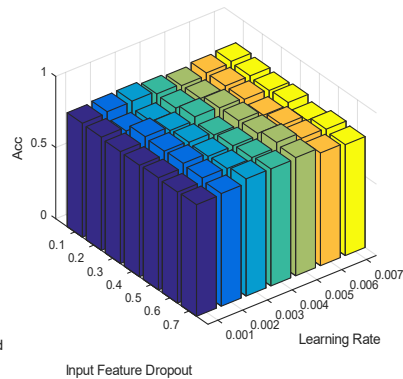

(f)

Supplement: S1 Raw images — (PDF) [file pone.0267565.s001.pdf]
